# Supplementary figures and images for: Dual targeting of histone deacetylases and MYC as potential treatment strategy for H3-K27M pediatric gliomas
Source: eLife. 2024 Aug 2;13:RP96257. doi: 10.7554/eLife.96257 (PMC11296706; doi:10.7554/eLife.96257)

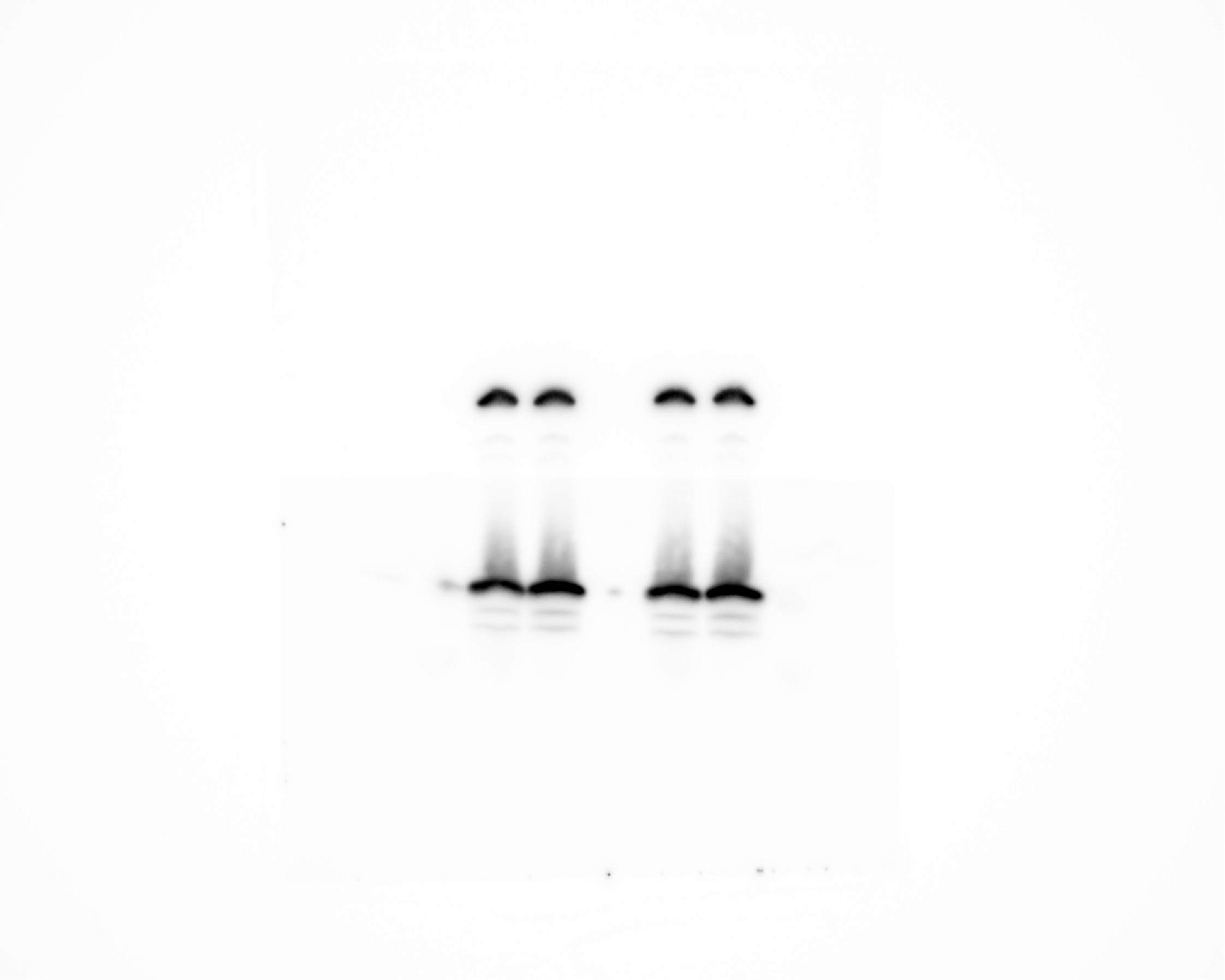

Supplement: Figure 1—figure supplement 1—source data 1. [file elife-96257-fig1-figsupp1-data1.zip › figure 1-figure supplement 1-source data 1/Panel S1C blot.tif]

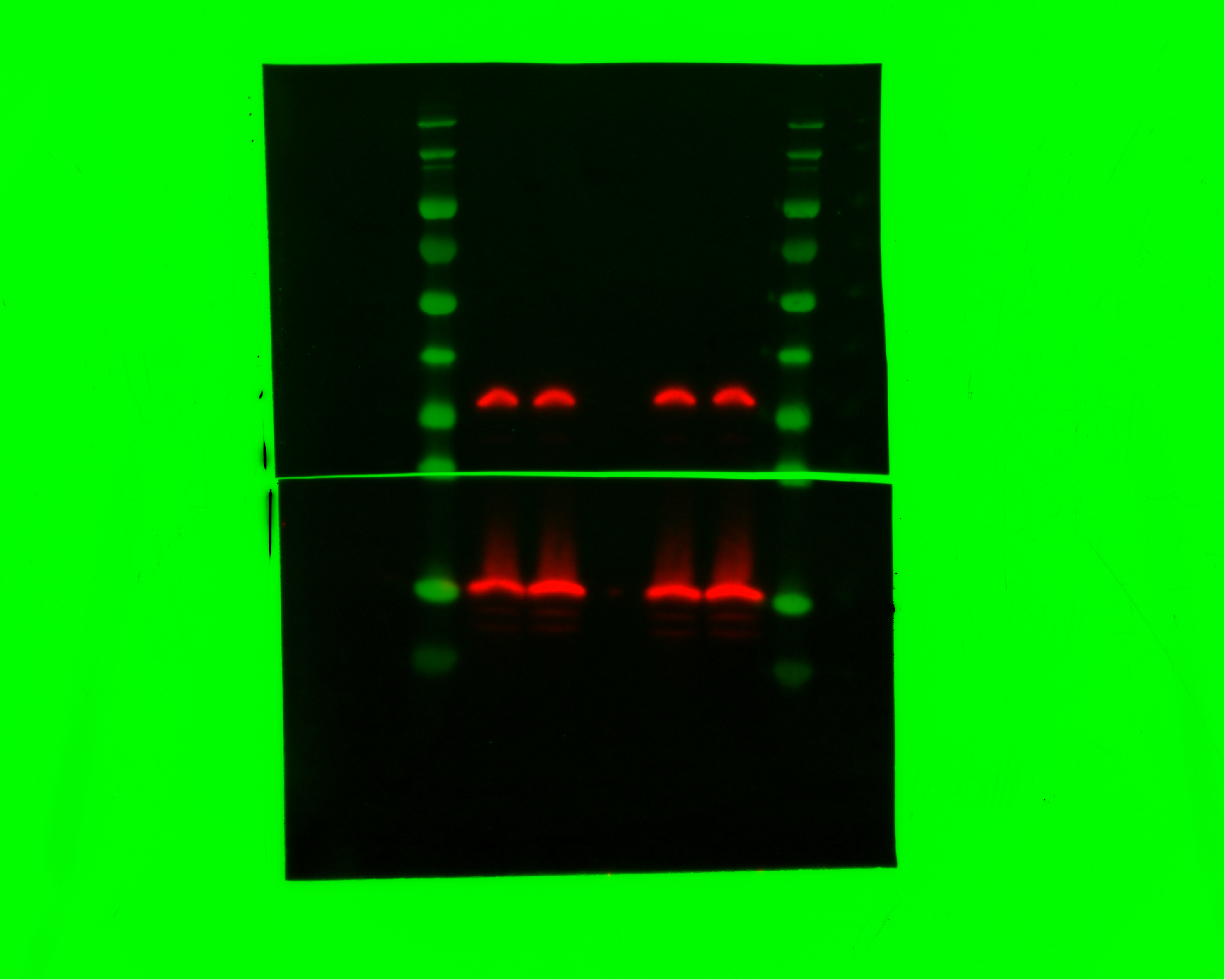

Supplement: Figure 1—figure supplement 1—source data 1. [file elife-96257-fig1-figsupp1-data1.zip › figure 1-figure supplement 1-source data 1/Panel S1C multi.tif]

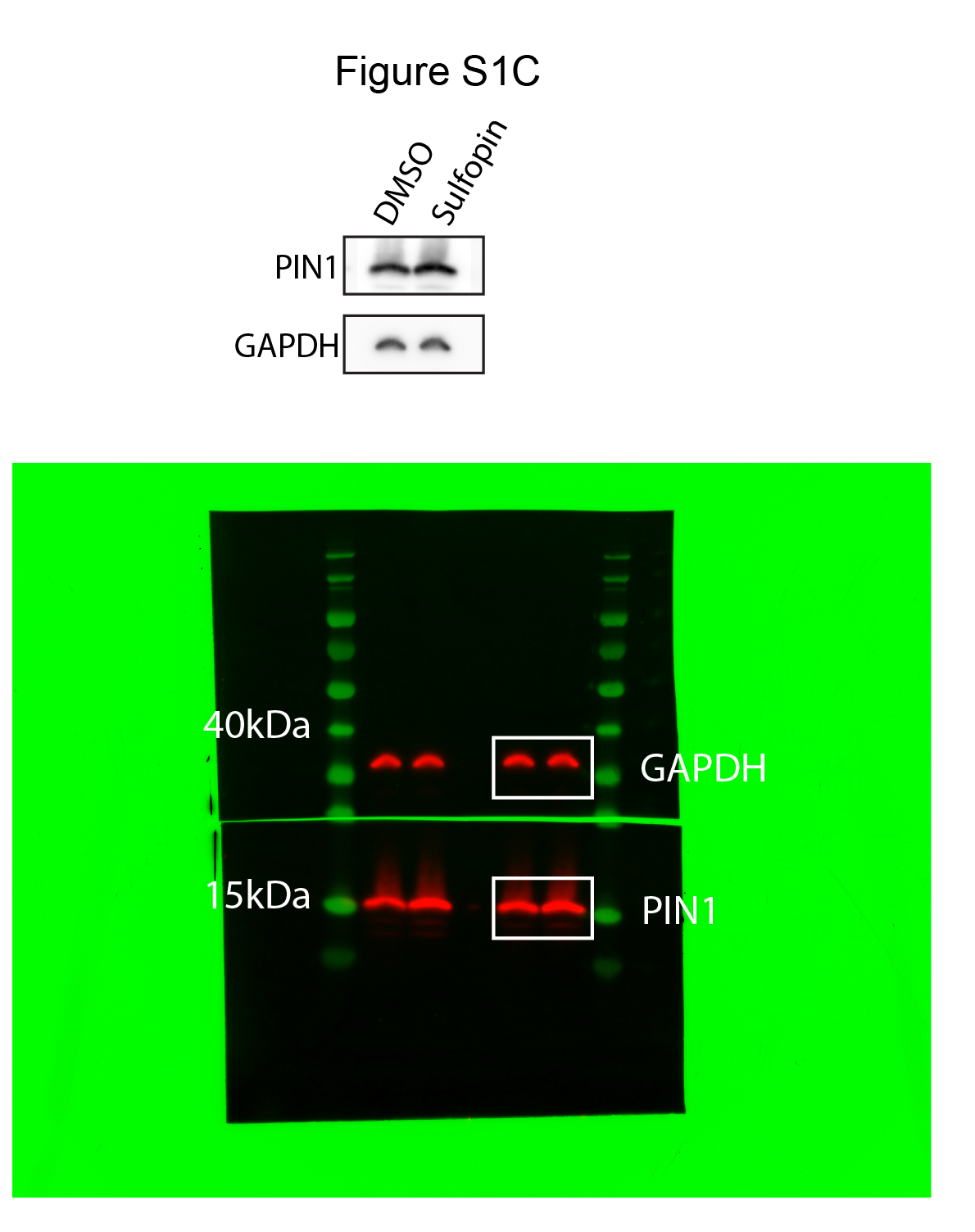

Supplement: Figure 1—figure supplement 1—source data 2. [file elife-96257-fig1-figsupp1-data2.zip › figure 1-figure supplement 1-source data 2/20231005_western_PIN1-02.png]

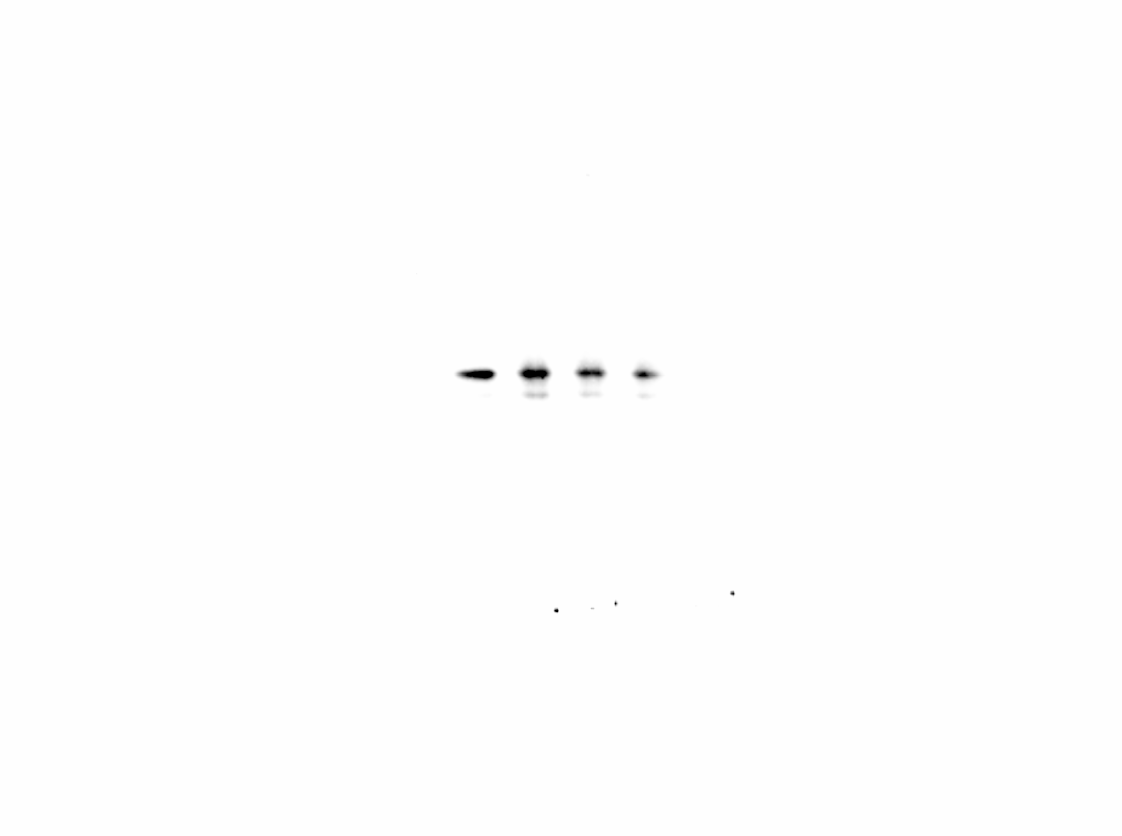

Supplement: Figure 2—source data 1. [file elife-96257-fig2-data1.zip › Figure2 -source data1/ATF3.tif]

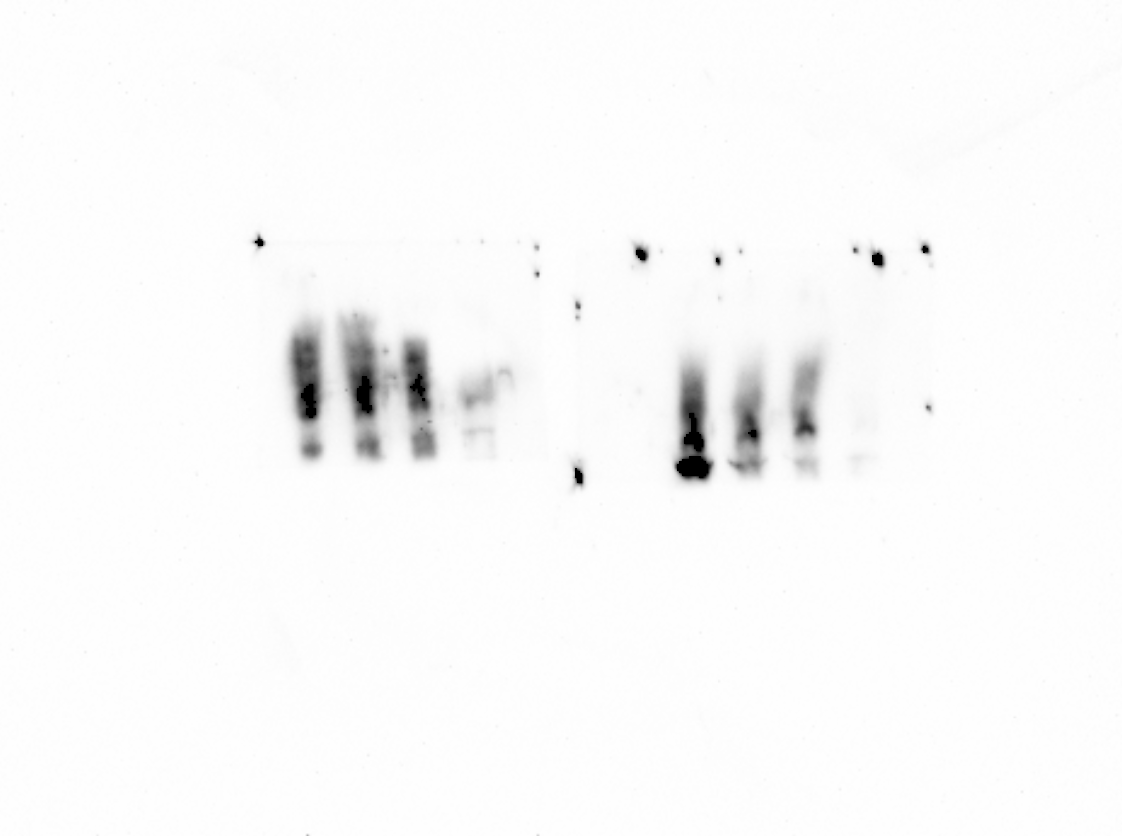

Supplement: Figure 2—source data 1. [file elife-96257-fig2-data1.zip › Figure2 -source data1/mTOR.tif]

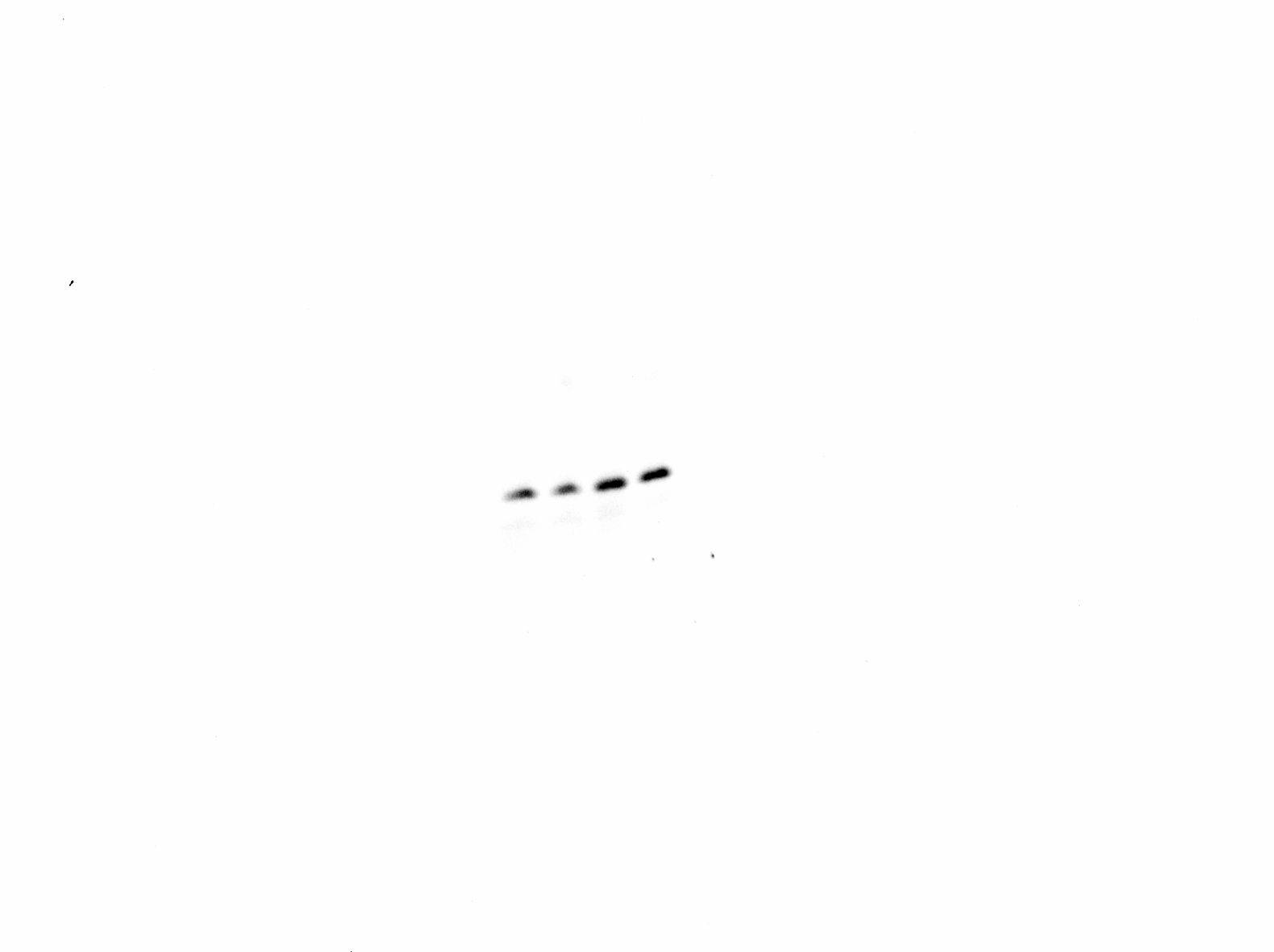

Supplement: Figure 2—source data 1. [file elife-96257-fig2-data1.zip › Figure2 -source data1/P21.tif]

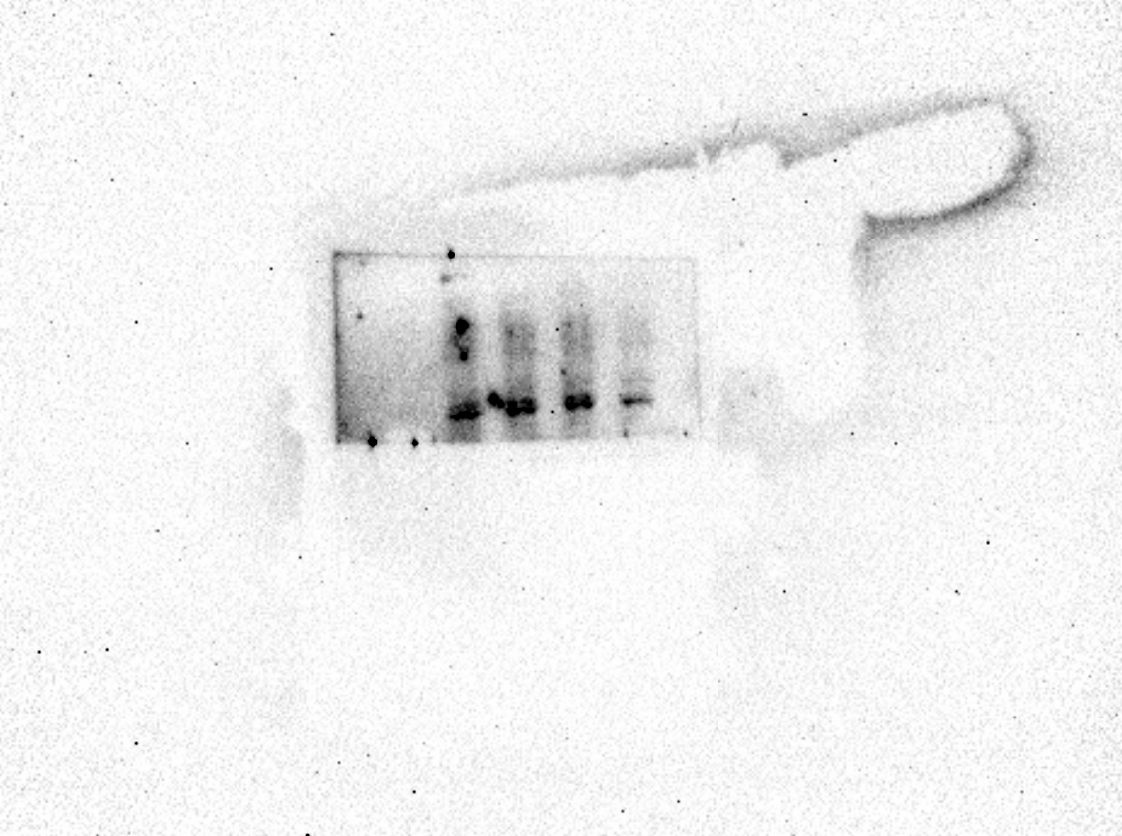

Supplement: Figure 2—source data 1. [file elife-96257-fig2-data1.zip › Figure2 -source data1/Pmtor.tif]

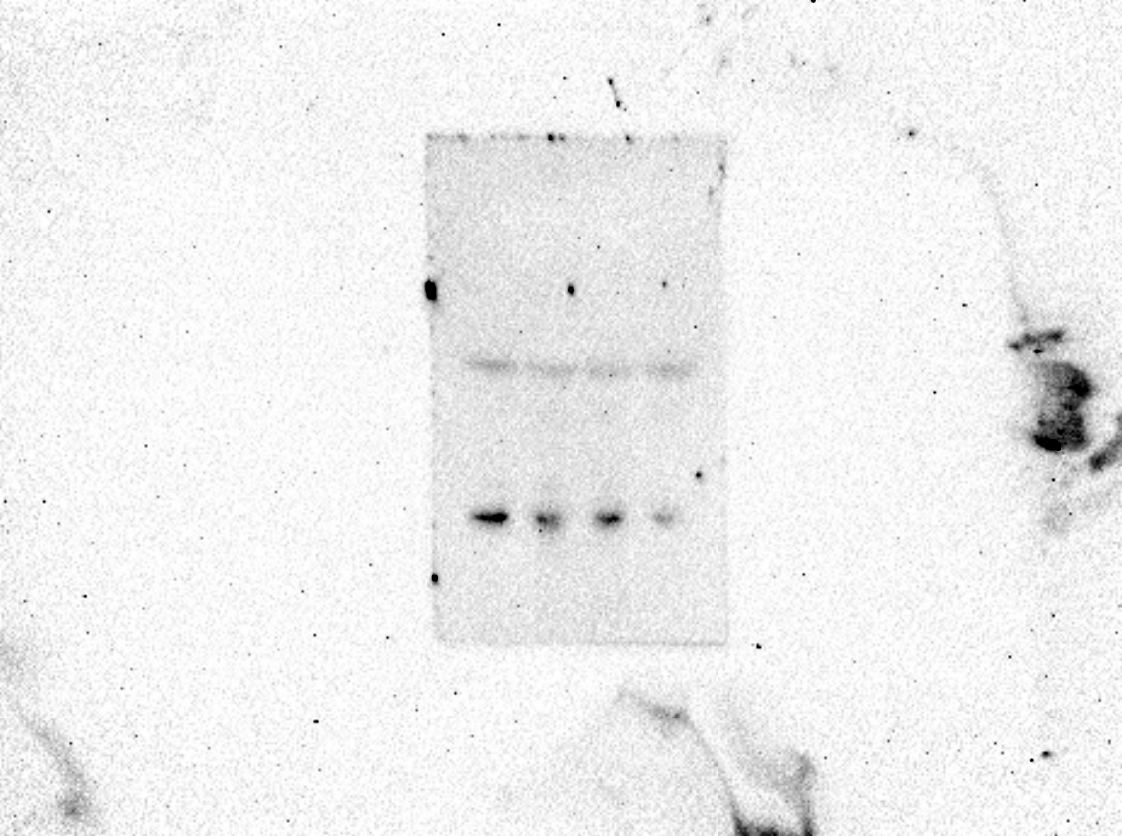

Supplement: Figure 2—source data 1. [file elife-96257-fig2-data1.zip › Figure2 -source data1/PS6.tif]

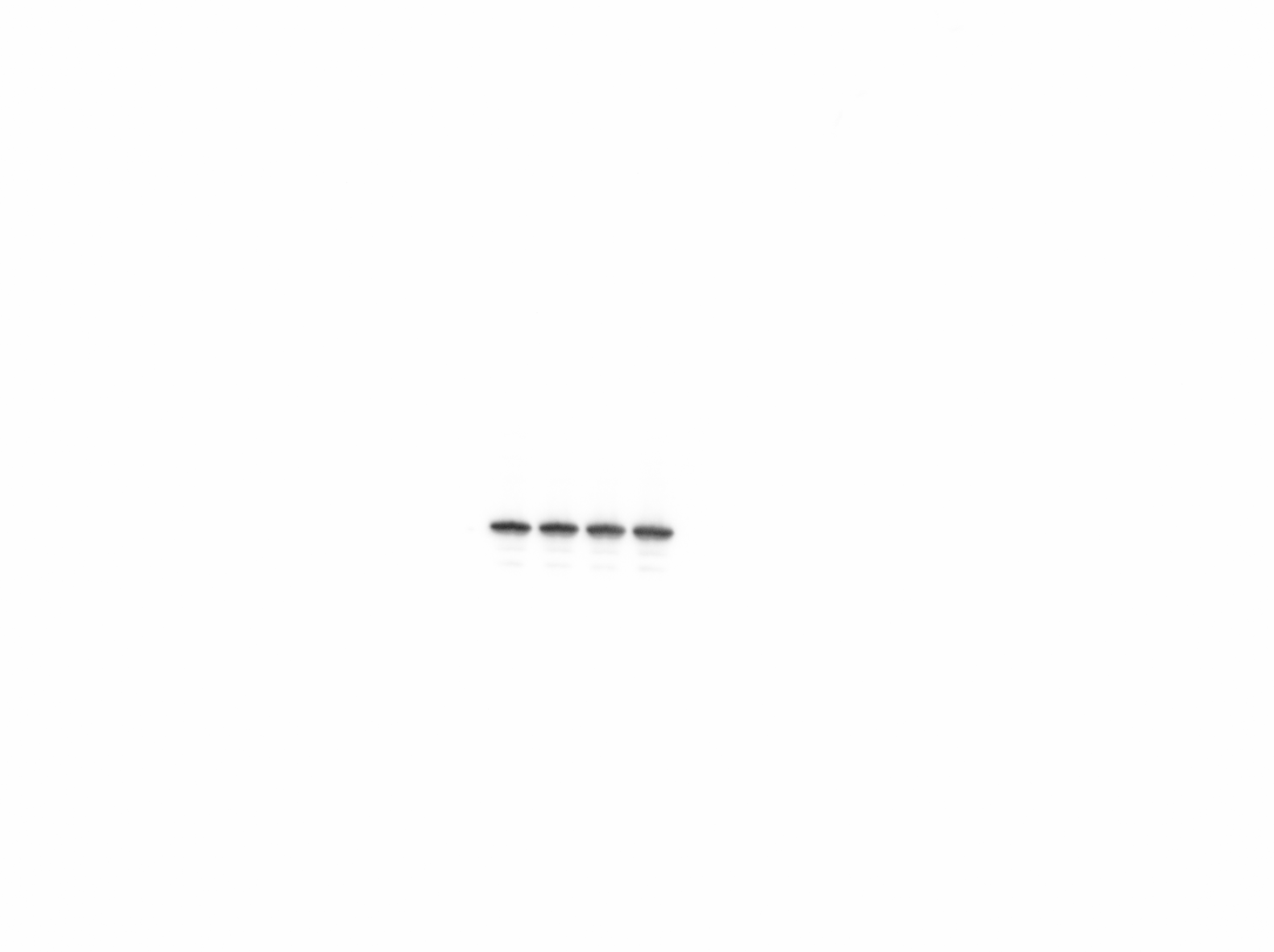

Supplement: Figure 2—source data 1. [file elife-96257-fig2-data1.zip › Figure2 -source data1/tubulin.tif]

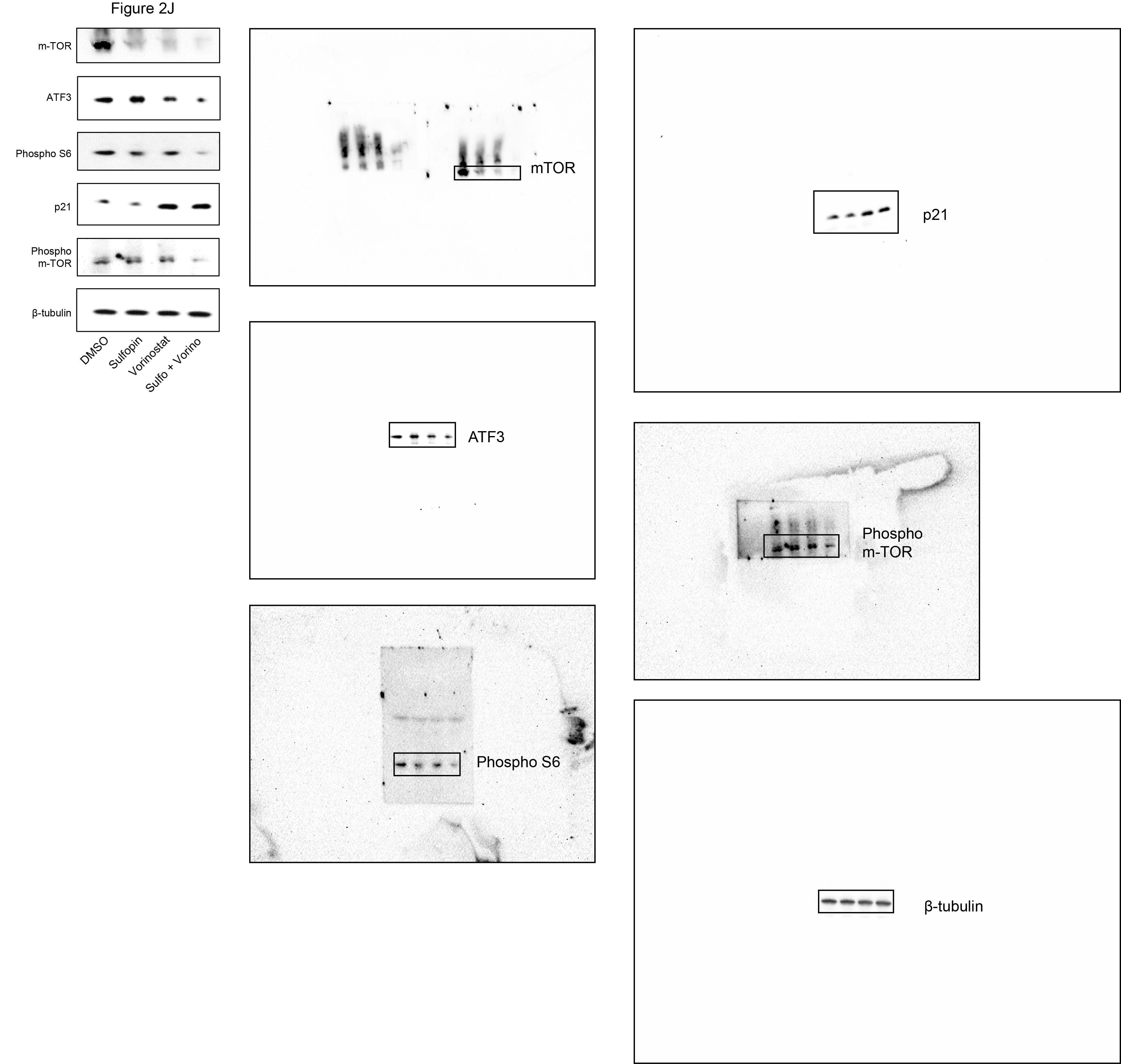

Supplement: Figure 2—source data 2. [file elife-96257-fig2-data2.zip › Figure2 -source data2/Figure2 - source datat2.png]

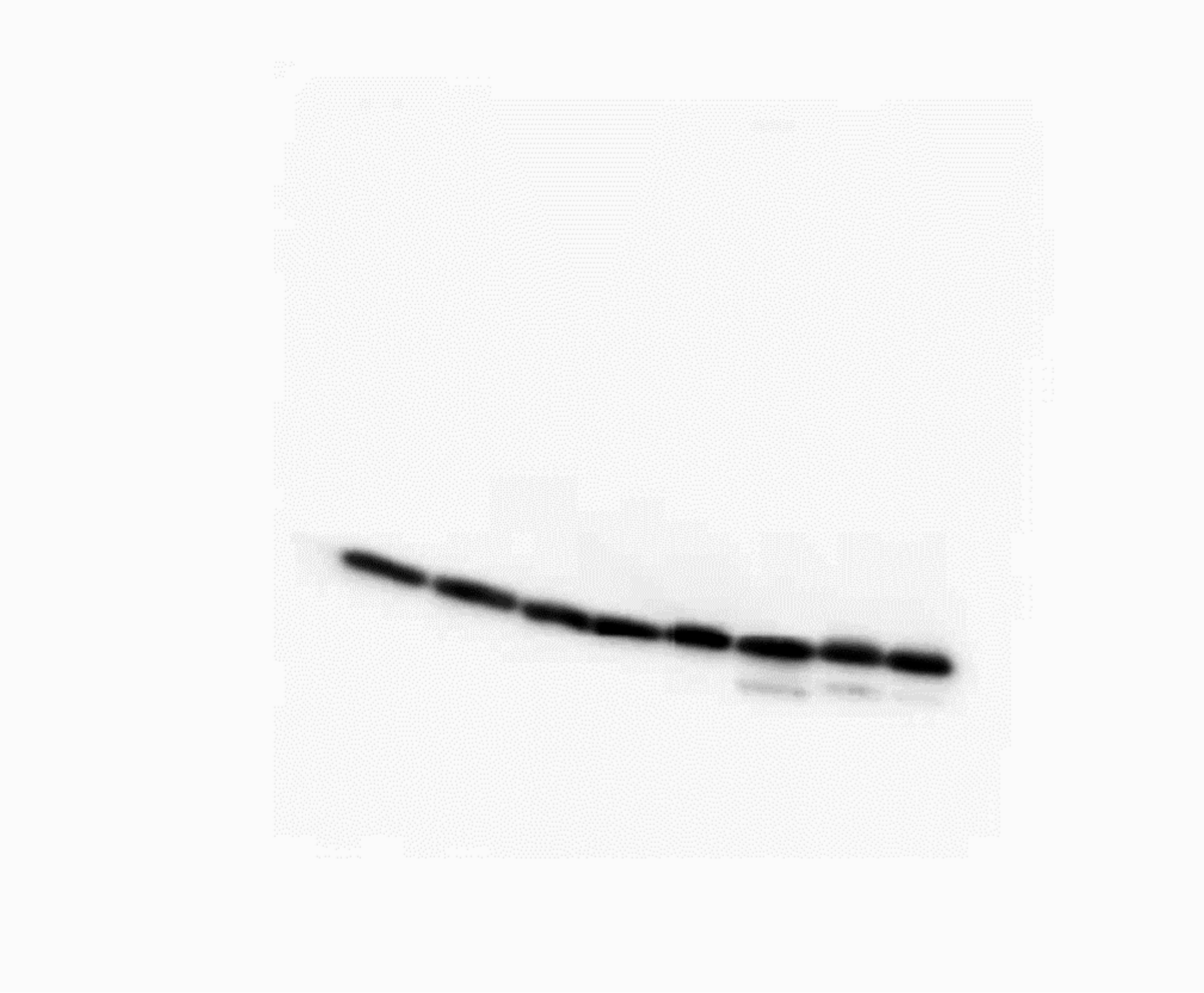

Supplement: Figure 3—source data 1. [file elife-96257-fig3-data1.zip › figure 3-source data 1/H3.tif]

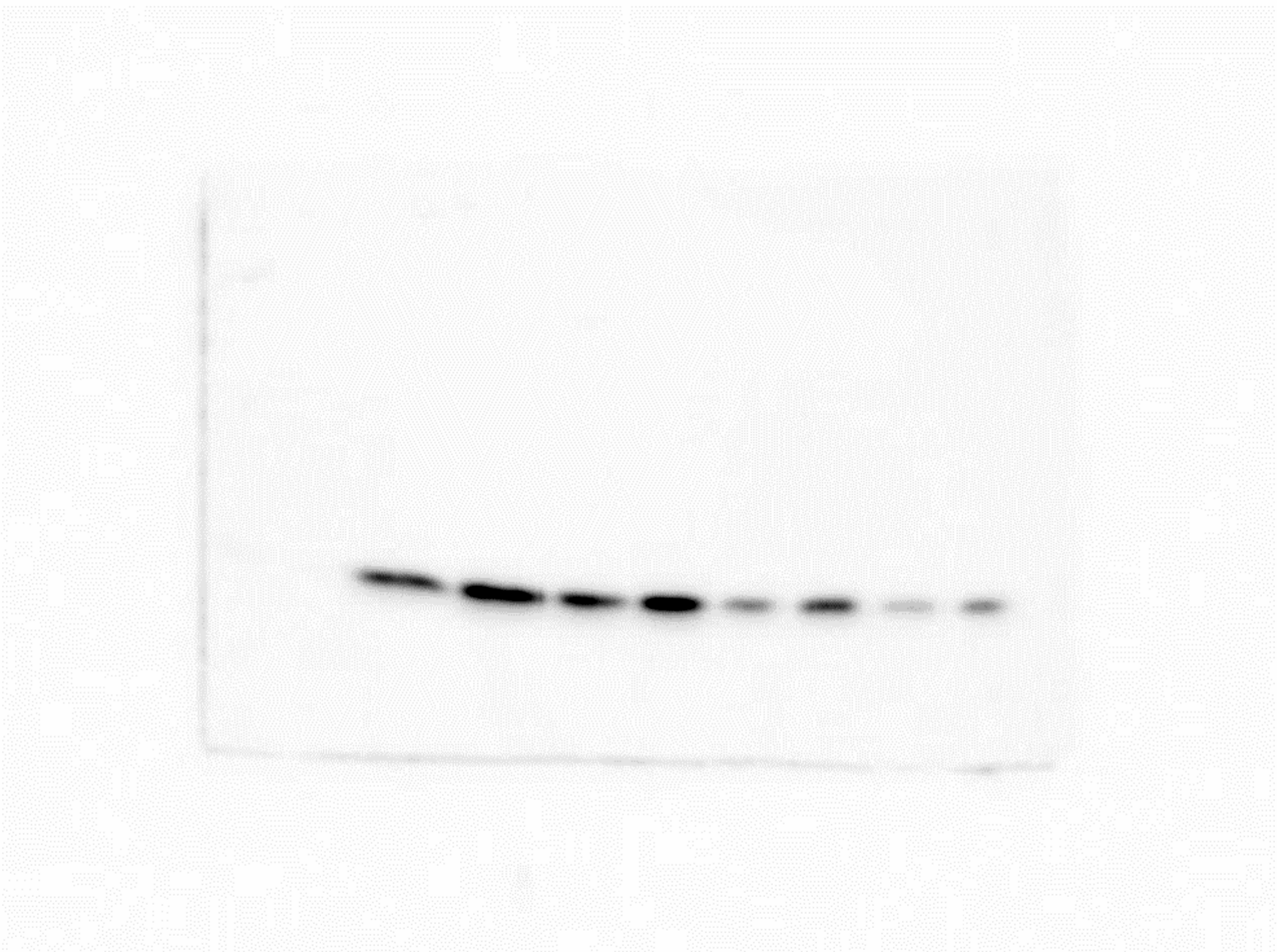

Supplement: Figure 3—source data 1. [file elife-96257-fig3-data1.zip › figure 3-source data 1/K27ac.tif]

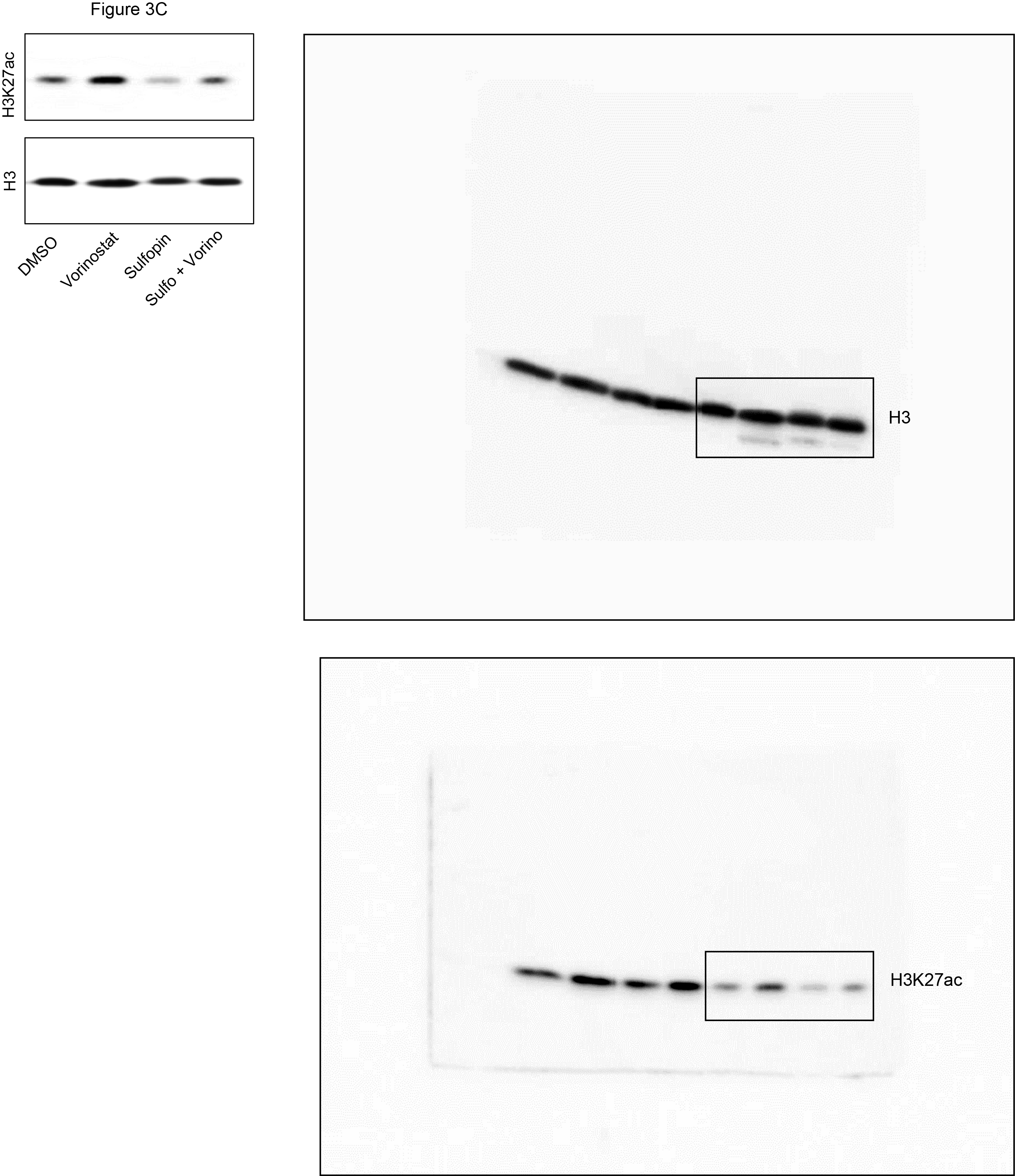

Supplement: Figure 3—source data 2. [file elife-96257-fig3-data2.zip › figure 3-source data 2/Figure3 - source datat2.png]
