# Supplementary material for: Dual targeting of histone deacetylases and MYC as potential treatment strategy for H3-K27M pediatric gliomas
Source: eLife. 2024 Aug 2;13:RP96257. doi: 10.7554/eLife.96257 (PMC11296706; doi:10.7554/eLife.96257)
Supplement: Supplementary file 2. [file elife-96257-supp2.docx]

Supplementary file 2 – Reagents and resources

| REAGENT or RESOURCE | SOURCE | IDENTIFIER |
| --- | --- | --- |
| Antibodies | | |
| Histone H3 (K27M Mutant Specific) (D3B5T) Rabbit mAb | Cell signaling | Cat#74829; RRID:AB_2799861 |
| Peroxidase-AffiniPure Goat Anti-Rabbit IgG | Jacksoon Immuno Research Laboratory | Cat#111-035-144; RRID:AB_2307391 |
| Acetyl-Histone H3 (Lys27) (D5E4) XP® Rabbit mAb | Cell signaling | Cat#8173; RRID:AB_10949503 |
| Acetyl-Histone H3 (Lys27) (D5E4) XP(R) Rabbit mAb (Alexa Fluor 647 Conjugate) | Cell signaling | Cat#39030; RRID:AB_2799145 |
| Tri-Methyl-Histone H3(K27) (C36B11) Rabbit mAb | Cell signaling | Cat#9733; RRID:AB_2616029 |
| Histone H3 (D1H2) XP® Rabbit mAb | Cell signaling | Cat#4499; RRID:AB_10544537 |
| mTOR (7C10) Rabbit mAb | Cell signaling | Cat#2983; RRID:AB_2105622 |
| p21 Waf1/Cip1 (12D1) Rabbit mAb | Cell signaling | Cat#2947; RRID: AB_823586 |
| Phospho-S6 Ribosomal Protein (Ser235/236) (D57.2.2E) XP® Rabbit mAb | Cell signaling | Cat#4858; RRID:AB_916156 |
| Phospho-mTOR (Ser2448) Antibody | Cell signaling | Cat#2971; RRID:AB_330970 |
| Recombinant Anti-ATF3 antibody [EPR19488] - ChIP Grade | Abcam | Cat#207434; RRID:AB_2734728 |
| Cy3 AffiniPure Donkey Anti-Rabbit IgG (H+L) | Jacksoon Immuno Research Laboratory | Cat#711-165-152; RRID:AB_2307443 |
| Monoclonal Anti-β-Tubulin I | Sigma | Cat#T7816-100UL; RRID:AB_261770 |
| PIN1 antibody | Cell signaling | Cat#3722; RRID:AB_10692654 |
| Anti-Glyceraldehyde-3-Phosphate Dehydrogenase, clone 6C5 (GAPDH) | Merck-Millipore | Cat#MAB374; RRID:AB_2107445 |
| Chemicals, Peptides, and Recombinant Proteins | | |
| pAG-MNase enzyme | Prof. Igor Ulitski | Addgene #123461 |
| Nextera Tn5 enzyme | Illumina | Cat#20034197 |
| Micrococcal Nuclease | ThermoFisher Scientific | Cat#88216 |
| Klenow Fragment 3'→5' exo- | NEB | Cat#M0212S |
| T4 Polynucleotide Kinase | NEB | Cat#M0201L |
| Neurobasal-A medium | Thermo Fisher | Cat#10888022 |
| DMEM-F12 | Thermo Fisher | Cat#11330032 |
| HEPES buffer | Thermo Fisher | Cat#15630056 |
| MEM sodium-pyruvate | Thermo Fisher | Cat#11360039 |
| MEM non-essential amino acids | Thermo Fisher | Cat#11140035 |
| Glutamax | Thermo Fisher | Cat#35050038 |
| antibiotic antimycotic | Thermo Fisher | Cat#15240096 |
| B-27 supplement w/o vitamin A | Thermo Fisher | Cat#12587010 |
| H-EGF | Shenandoah | Cat#100-26 |
| H-FGF-basic-154 | Shenandoah | Cat#100-146 |
| H-PDGF-AA | Shenandoah | Cat#100-16 |
| H-PDGF-BB | Shenandoah | Cat#100-18 |
| Heparin sodium salt from porcine intestinal mucosa | Merck | Cat#H3149; CAS#9041-08-1 |
| TrypLE Express | Thermo Fisher | Cat#12604013 |
| protease inhibitors | Sigma | Cat#P8340 |
| Sodium butyrate | Sigma | Cat#303410 |
| Fluorescently labeled dATP | Jena Bioscience | Cat#NU-1611-Cy3/Cy5 |
| Biotinylated dUTP | Jena Bioscience | Cat#NU-803-BIOX |
| Alconox | Sigma | Cat#242985 |
| KOH | Sigma | Cat#484016 |
| HPLC EtOH | J.T baker | Cat#8462-25 |
| 3-Aminopropyltriethoxysilane | ACROS Organics | Cat#430941000 |
| Biotin-PEG | Laysan | Cat#SVA-5000 |
| mPEG | Laysan | Cat#MPEG-SVA-5000 |
| Streptavidin | SIGMA | Cat#S4762 |
| TetraSpeck beads | ThermoFisher Scientific | Cat#T7279 |
| MES pH 6.5 | Boston Bioproducts Inc | Cat#NC9904354 |
| Sulfopin | Prof. Nir London | N/A |
| Vorinostat | MCE | Cat#HY-10221 |
| Vorinostat | LC | Cat#V-8477 |
| MM-102 | Selleck | Cat#S7265 |
| EPZ6438 | Selleck | Cat#S7128 |
| GSK-J4 | Selleck | Cat#S7070 |
| RPMI media | Biological Industries | Cat#01-101-1A |
| Fetal Bovine Serum (FBS) | Biological Industries | Cat#04-001-1A |
| L-glutamine | Biological Industries | Cat#03-020-1A |
| penicillin/streptomycin solution | Biological Industries | Cat#03-031-1B |
| M-MLV-RT | Promega | Cat#M1701 |
| KAPA SYBR FAST mix | Kapa Biosystems | Cat#KK4660 |
| Laemmli sample buffer | Bio-Rad | Cat#1610747 |
| DTT | Promega | Cat#V3151 |
| Tris-Glycine 4-20% gel | ThermoFisher Scientific | Cat#XP04205BOX |
| TG-SDS buffer | Bio-rad | Cat#1610732 |
| TBS buffer | Bio-rad | Cat#1706435 |
| Tween20 | Sigma | Cat#P1379 |
| Xylene | Bio-lab | Cat#242500 |
| normal horse serum | Vector Laboratories | Cat#EW-93951-74 |
| triton | Sigma | Cat#9036-19-5 |
| Hoechst | ThermoFisher scientific | Cat#H3570 |
| Aqua-Poly/Mount | Polysciences | Cat#18606 |
| OPAL 650 | Akoya | FP1496001KT |
| OPAL 570 | Akoya | FP1488001KT |
| D-luciferin | Perkin-elmer | Cat#122799 |
| Concanavalin A-coated beads | Bangs Laboratories | Cat#BP531 |
| ECL Detection Reagent | Bio-rad | Cat#1705061 |
| EG400 | Sigma | Cat#25322-68-3 |
| Critical Commercial Assays | | |
| NextSeq 500/550 mid Output Kit v2.5 (150 Cycles) | Illumina | Cat#20024904 |
| CelltiterGlo | Promega | Cat#G7571 |
| Nucleospin gel and PCR clean-up kit | Machery-Nagel | Cat#740609 |
| NextSeq 500/550 High Output Kit v2.5 (75 Cycles) | Illumina | Cat#20024906 |
| NucleoSpin RNA, Mini kit for RNA purification | Machery-Nagel | Cat#740955.50 |
| Trans-Blot Turbo Mini | Bio-rad | Cat#1704156 |
| Deposited Data | | |
| Cut&Run and MARS-seq data | This study | GSE221614 |
| Gene expression data of WT vs. K27M DMG tumor samples | Mackay et al., 2017 | https://pedcbioportal.kidsfirstdrc.org/ |
| Gene expression data of DMG tumors vs. normal samples | Berlow et al., 2018 | S2 Table |
| Experimental Models: Cell Lines | | |
| SU-DIPG6 | Michelle Monje's lab, Stanford University | RRID CVCL_IT40 |
| SU-DIPG13 | Michelle Monje's lab, Stanford University | RRID CVCL_IT41 |
| SU-DIPG25 | Michelle Monje's lab, Stanford University | RRID:CVCL_C1N0 |
| SU-DIPG38 | Michelle Monje's lab, Stanford University | RRID:CVCL_C1N6 |
| SU-DIPG36 | Michelle Monje's lab, Stanford University | RRID:CVCL_C1N5 |
| SU-DIPG17 | Michelle Monje's lab, Stanford University | RRID:CVCL_C1MW |
| SU-DIPG50 | Michelle Monje's lab, Stanford University | N/A |
| SU-DIPG21 | Michelle Monje's lab, Stanford University | RRID:CVCL_C1MX |
| SU-DIPG13P* | Michelle Monje's lab, Stanford University | N/A |
| SU-DIPG6-GFP | Michelle Monje's lab, Stanford University | N/A |
| CRL-1718 | Daniel Michaelson lab, Tel-Aviv University, Israel | RRID:CVCL_1118 |
| SU-DIPGXIII K27M | Nada Jabado's lab, McGill University | N/A |
| SU-DIPGXIII K27M KO c5 | Nada Jabado's lab, McGill University | N/A |
| BT245 K27M | Nada Jabado's lab, McGill University | N/A |
| BT245-KO | Nada Jabado's lab, McGill University | N/A |
| Experimental Models: Organisms/Strains | | |
| NGS mice (NOD-SCID-IL2R gamma chain-deficient) | Jackson | Cat#005557 |
| Oligonucleotides | | |
| random hexamer primers | Thermo Scientific | Cat#SO142 |
| PES1 qPCR primers FWD:TCTTCCTGTCCATCAAAGGC REV:GTGGCCATGACCCTGTAGTC |  |  |
| HSP90AB1 qPCR primers FWD:CCAGGCACTTCGGGACAACTC REV:CAAGGGAAAAGCCAGAAGATAGCA |  |  |
| NCL qPCR primers FWD:ACTGACCGGGAAACTGGGTC REV:TGGCCCAGTCCAAGGTAACT |  |  |
| E2F2 qPCR primers FWD:ACAAGGCCAACAAGAGGCTG REV:TCAGTCCTGTCGGGCACTTC |  |  |
| RNA5S1 qPCR primers FWD:GGCCATACCACCCTGAACGC REV:CAGCACCCGGTATTCCCAGG |  |  |
| RNA5-8SN4 qPCR primers FWD:GCTCTAACCTTACCTACCTGG REV:TGAGCCATTCGCAGTTTCAC |  |  |
| MYC qPCR primers FWD:CAGCTGCTTAGACGCTGGATT REV:GTAGAAATACGGCTGCACCGA |  |  |
| mTOR qPCR primers FWD:TATCCGCTACTGTGTCTTGGC REV:CTCTGTCAGGATCTGGATGAGC |  |  |
| PSMD3 qPCR primers FWD:GAGTTCCTGGACAAGCTGGA REV:AGGTAATTCCGCAGCAGGAG |  |  |
| NOC4L qPCR primers  FWD: GCCACCCCTCCTTTCAGG  REV: GGGGAACAGGTAGTTGCCTT |  |  |
| GAPDH:  FWD: GGTGTGAACCATGAGAAGTATGA  REV: GAGTCCTTCCACGATACCAAAG |  |  |
| HPRT:  FWD: GGTCCTTTTCACCAGCAAGCT  REV: TGACACTGGCAAAACAATGCA |  |  |
| Software and Algorithms | | |
| ImageJ | NIH | RRID: SCR_00307 |
| Fiji | NIH | RRID: SCR_002285 |
| Cellprofiler | Broad Institute | RRID:SCR_007358 |
| cutadapt | (Martin, 2011) | https://cutadapt.readthedocs.io/en/stable/ |
| Bowtie version 2.3.5.1 | (Langmead and Salzberg, 2012) | http://bowtie-bio.sourceforge.net/bowtie2/index.shtml |
| FastQC | Andrews et al. 2010) | https://www.bioinformatics.babraham.ac.uk/projects/fastqc/ |
| MACS2 | (Zhang et al., 2008) | https://github.com/macs3-project/MACS |
| Picard toolkit | Broad Institute | https://broadinstitute.github.io/picard/ |
| deepTools2 suite | (Ramírez et al., 2016) | https://deeptools.readthedocs.io/en/develop/index.html |
| IGV (2.8.6) | Broad Institute | RRID:SCR_011793 |
| ChIPseeker | (Yu et al., 2015) | https://bioconductor.org/packages/release/bioc/html/ChIPseeker.html |
| GREAT | (McLean et al., 2010) | http://great.stanford.edu/public/html/ |
| ngs.plot | (Shen et al., 2014) | RRID:SCR_011795 |
| HOMER | (Heinz et al., 2010) | http://homer.ucsd.edu/homer/motif/ |
| UTAP transcriptome analysis pipeline | (Kohen et al., 2019) | N/A |
| HTSeq-count | (Anders et al., 2014) | https://htseq.readthedocs.io/en/release_0.11.1/count.html |
| DESeq2 | (Love et al., 2014) | https://bioconductor.org/packages/release/bioc/html/DESeq2.html |
| bedtools/2.26.0 | (Quinlan and Hall, 2010) | <https://bedtools.readthedocs.io/en/latest/index.html> |
| Gene set enrichment analysis (GSEA) | (Subramanian et al., 2005; Mootha et al., 2003) | https://www.gsea-msigdb.org/ |
| Enrichr | (Kuleshov et al., 2016) | https://maayanlab.cloud/Enrichr/ |
| gProfiler | (Raudvere et al., 2019) | https://biit.cs.ut.ee/gprofiler/gost |
| SynergyFinder 2.0 | (Yadav et al.,2015) | http://www.synergyfinder.org/ |
| GeneHancer database v5.9 | (Fishilevich et al., 2017) | https://genome.ucsc.edu/cgi-bin/hgTrackUi?db=hg19&g=geneHancer |
| QuPath | (Bankhead et al., 2017) | N/A |
| StarDist | (Schmidt et al., 2018) | N/A |
